# Acrylamide and Thermal-Processing Indexes in Market-Purchased Food

**DOI:** 10.3390/ijerph16234724

**Published:** 2019-11-27

**Authors:** Joanna Michalak, Marta Czarnowska-Kujawska, Elżbieta Gujska

**Affiliations:** Department of Commodity Science and Food Analysis, Faculty of Food Science, University of Warmia and Mazury in Olsztyn, 10-693 Olsztyn, Poland; seniutaj@uwm.edu.pl (J.M.); elka@uwm.edu.pl (E.G.)

**Keywords:** acrylamide, thermal-processing indexes, HMF, surface colour, Maillard reactions, caramelization, market-purchased food, food safety, food composition, food analysis

## Abstract

Determining acrylamide (AA) content in foods using chromatographic methods is expensive and time-consuming. Therefore, there is a need to develop a simple, economical method for monitoring the content of acrylamide in foods. This study analysed whether there is a relationship between acrylamide levels with some heat-induced parameters, such as 5-hydroxymethylfurfural (HMF) and browning, in order to assess their usefulness in predicting the potential acrylamide levels in market-purchased food. Sixty plant-based food products were tested. The correlation coefficients for AA levels with L*, a* and b* values and HMF content were significant (*p* < 0.05) for French fries and potato chips. There was no statistically significant correlation between thermal-processing indexes (HMF and colour parameters) and acrylamide levels in commercial bread, breakfast cereals and biscuits. The results indicate that these classical thermal-processing indexes are not directly related to the acrylamide content in commercial cereal-based food and they cannot be indicators of AA level. Thus, the correlation between HMF and colour parameters with acrylamide content depends on the type of food and it is difficult to estimate the amount of AA based on these classical thermal-processing indexes of market-purchased food.

## 1. Introduction

One of the latest neurotoxic and carcinogenic substances discovered in food is acrylamide (AA). Acrylamide is primarily found in heated plant-based foods such as potato, cereal and bakery products [1]. The main pathway for AA formation in foods is the Maillard reaction, but there are also other less significant pathways of its formation. Acrylamide can be formed in heated food by the conversion of acrolein, acrylic acid, wheat gluten or by de-amination of 3-aminopropionamide or as a result of asparagine enzymatic decarboxylation [2,3,4]. Estimation of acrylamide occurrence in food commodities is a great concern in many countries. Factors such as differences in food composition in terms of carbohydrate, free asparagine, reducing sugars, ammonium bicarbonate, competing amino acids and water contents, together with temperature (more than 120 °C) and pH levels, can influence the acrylamide amount [5,6]. In addition, the quantification of acrylamide in foods is a challenge due to its low molecular weight, high polarity, very good solubility in water, high reactivity and low volatility. The main problem for quantification in complex systems, such as food, are compounds that interfere with detection due to the matrix effect, and their losses during extraction and sample preparation [3,7]. At present, two techniques are used: gas chromatography (GC) and liquid chromatography (LC), both coupled with mass spectrometry (MS), while many others have been developed [3]. The European Committee for Standardization, CEN, has developed standard methods for both techniques: EN 16618:2015 Food analysis—Determination of acrylamide in food by liquid chromatography-tandem mass spectrometry (LC-ESI-MS/MS) and PREN 16987:DRAFT 2016 Foodstuffs—Determination of acrylamide in coffee and coffee products by HPLC-MS/MS and GC-MS [8,9]. However, all of these methods are severely inconvenient, expensive and time-consuming and some of them show various limitations in the routine acrylamide analysis for a wide range of food products. Therefore, there is a need to develop a fast and economical method for controlling the content of acrylamide in foods.

Another compound, besides acrylamide, formed during the Maillard reactions is 5-hydroxymethylfurfural (HMF), which can be also formed as a result of hexoses dehydration under mildly acidic conditions (caramelisation reaction). Both reactions occur on the food surface during thermal treatment and lead to the production of desirable colour, flavour and aroma. The typical brown colour is attributed to the production of polymeric products during both reactions and HMF and furfural have been indicated as precursors of such polymers [10,11,12]. HMF is especially present in over-processed foods but there are no maximum values prescribed for most food products [3]. However, according to European Food Safety Authority (EFSA) recommendations, the specifications defined for caramel colours in EU legislation should be updated to also include maximum levels for HMF [13]. In addition to temperature as the main factor, the rate of HMF formation in food also depends on the type of sugar, pH, water activity and the proportion of divalent cations [3]. Caramelization requires higher temperatures than the Maillard reaction [14], and the type and amount of sugars have a most significant impact on the HMF formation [15]. High levels of HMF can be found in dried fruits (e.g., 25–2900 mg/kg), coffee (e.g., 100–4100 mg/kg) and caramel products [5]. HMF has been considered a heat-induced marker for a wide range of carbohydrate-containing foods and it is used for monitoring the heating process applied to milk products, cereal products such as pasta drying, cookies, bread baking, as well as extruded baby cereals, breakfast cereals and toasted bread [1,5,10,16]. On the other hand, HMF has also been extensively investigated as a food contaminant due to its toxic, mutagenic and carcinogenic effects on the human body [1,5,10]. The literature describes different methods for the HMF determination, which can be classified into three main groups: colorimetric, spectrophotometric and chromatographic methods. High-performance liquid chromatography (HPLC) with a UV detector is the most commonly used HMF determination in numerous food products [3].

Colour is considered one of the most important quality parameters of food products and is critical in the consumer’s acceptance. Many studies have found a correlation between browning colour and AA formation in laboratory-processed food products [17,18,19,20]. However, few studies are concerned with the relationship between AA content with the colour of market-purchased food. Traditionally, measurements of colour and 5-hydroxymethylfurfural content have been applied as chemical indexes of thermal processing in different products. Analytical methodologies for HMF analysis and product browning are well-established. However, acrylamide determination in foods requires more sophisticated laboratory equipment. It is also important to carry out the study on real commercial samples since both ingredients, HMF and AA, and processing conditions are not sufficiently well-controlled. Therefore, the purpose of the present investigation was to study the relationship between acrylamide levels with some heat-induced parameters such as browning and HMF content in order to assess their usefulness for predicting the potential acrylamide levels in some commercial plant-based products.

## 2. Materials and Methods 

### 2.1. Food Samples

The study was conducted on food products with high acrylamide formation, such as potato-derived foodstuffs, or with significant impact on the dietary habits of the population, like cereal-based foodstuffs. Sixty different market-purchased food products were tested. French fries ready-to-eat were purchased from a series of fast-food services. Other products were purchased from different supermarkets. The research materials were divided into six food groups including: French fries ready-to-eat (10 mm straight cut fries) (10), classic salted potato crisps (10), soft wheat bread (100% wheat flour) (10), crisp wheat bread (100% wheat flour) (10), breakfast cereals (100% wheat flakes) (10) and biscuits (100% wheat flour) (10). The colour analysis was carried out immediately after purchase. Next, all samples were ground and mixed in a blender to assure a homogeneous distribution of potential hotspots. 

### 2.2. Chemicals

The acrylamide standard (99.8%, catalogue No. 23701), 5-hydroxymethylfurfural (≥98.0%, catalogue No. 53407) and all chemicals of HPLC analytical grade were obtained from Sigma–Aldrich (St. Louis, MO, USA) and Merck (Darmstadt, Germany).

### 2.3. Colour Analysis

The surface colour of food products was measured by a portable spectrophotometer MiniScan EZ (HunterLab, Murnau, Germany) in units CIE L*a*b*, where L* represents lightness ranges from 0 (black) to 100 (white), positive a* = red, negative a* = green, positive b* = yellow and negative b* = blue. The colour was measured at ten different positions on the product surface. 

### 2.4. Determination of Acrylamide and 5-Hydroxymethylfurfural Content

The AA was determined with the method developed by Michalak et al. [7] using a Shimadzu LC–10A chromatograph equipped (Kyoto, Japan). Acrylamide quantification was performed using ion-pair RP-HPLC-DAD by comparison with the external acrylamide standard. UV absorbance spectra showed that the maximal absorption wavelength of acrylamide is 200 nm. The spectra of acrylamide in the sample were identical to that of standard, indicating that the hydrophobic interferences did not interfere with acrylamide determination in sample at 200 nm. The method was validated and tested for acrylamide determination in different market-purchased food products such as French fries, potato crisps, biscuits and breakfast cereals, including food difficult matrixes. In order to confirm acrylamide analysis LC–MS technique was used. The results of some tested samples were comparable with using an LC–MS technique. It was confirmed that the method used to determine acrylamide meets the performance criteria recommended by European Commission (EC) in the Commission Recommendation 2017 [21]. 

The HMF was determined by the procedure of Ferrer et al. [22] and Chavez-Servin et al. [23] using an Agilent 1200 chromatograph equipped (Santa Clara, CA, USA).

### 2.5. Statistical Analysis

For AA and HMF content, each sample was analysed in triplicate. The colour indexes measurements were performed in ten replicates. The results were presented as mean values ± standard deviations (SDs) of ten independent samples (*n* = 10). The data were analysed using the Statistica 12.5 software package (StatSoft, Poland). Significant differences were calculated using Duncan’s Multiple range test and were considered statistically significant at the 5% level. Correlations among AA, HMF and the colour parameters were determined by Pearson’s correlation analysis at the *p* < 0.05 confidence level.

## 3. Results and Discussion

In studies conducted worldwide, AA content in food ranged widely from lower than 100 μg/kg in high protein foods, to the highest amounts of 100–4000 μg/kg in high carbohydrate foods [5]. In the current study, the acrylamide content ranged from 55 μg/kg for soft wheat bread to 546 μg/kg for potato crisps (Table 1). Moreover, the results show great variability in acrylamide levels between different brands of the same product. The difference in food composition and in the processing conditions applied can easily explain the observed variability. 

In 2017, the European Commission (EC) adopted new reference values for the acrylamide content in the major food contributors to the intake of this compound [21]. In cases where the level of acrylamide in a foodstuff exceeds the acrylamide benchmark levels set by EFSA, investigations into the production and processing methods used by food producers must be carried out. According to the EC, in this way, the risks associated with the acrylamide intake can be regulated and, consequently, controlled. The benchmark levels established in 2017 [21] have been significantly lowered compared to the recommended levels in 2011 [24] and 2013 [25]. This was due to the fact that in recent years, monitoring has shown a decrease in acrylamide content in certain food categories, e.g., soft bread and potato products [24,25,26]. As shown in Table 1, the mean acrylamide level for half of the product groups was higher than benchmark levels for AA established by EC (2017/2158/EU) [21]. In the case of soft wheat bread, breakfast cereals and biscuits, the mean acrylamide contents were 10%, 34% and 49% higher than benchmark levels of 2017/2158/EU [21], respectively. In the present study, 30% of the tested bread, 80% of breakfast cereals and 100% of biscuits exceeded benchmark levels. In a study carried in Italy, Capei et al. [27] also found that 33% of breakfast cereals and 22.7% of biscuits exceeded the AA benchmark levels recommended by the EU in 2013 [25]. It seems that the mitigation measures taken by industry to reduce the levels of acrylamide are insufficient or ineffective. This means that additional efforts should be implemented by these companies to decrease acrylamide levels.

Browning is the final step of both the Maillard reaction and caramelization. The colour index L* ranged between 48.9 and 66.1 for crisp bread and French fries respectively (Table 1). All French fries were significantly brighter (*p* < 0.05) than other products, as indicated by the higher L* value. The colour analysis indicated that the crisp bread samples were redder than other samples. The value of a*, which is a measurement of the red colour, was 3.7 for French fries and 27.1 for crisp bread. The value of b*, an indicator of the yellow colour, ranged from 15.1 (crisp bread) to 20.6 (breakfast cereals). The correlation coefficients for AA with L*, a* and b* values were significant (*p* < 0.05) for market-purchased potato products, but not significant (*p* > 0.05) for bread, breakfast cereals or biscuits (Table 2). Many cereal-based products had darker colour (low lightness and high redness or yellowness) at lower AA contents, but others had brighter colours at higher AA contents. As far as colour results with the potato products, French fries with higher acrylamide levels generally had more red and darker colours, especially showing lightness (L*) decrease and redness (a*) increase. The highest levels of acrylamide were observed in potato chips, which had more yellow and darker colour, showing mainly lightness (L*) decrease and yellowness (b) increase. These results showed that the degree of surface browning could be used as an indicator of the acrylamide content in market-purchased potato products. This is in contrast to research of Serpen and Gökmen [28] who found that CIE b* values had lack of correlation with acrylamide (r = 0.2612) in potato crisps. However, it is known that many factors affect AA formation. These could be for example different reducing sugar concentration in raw materials used in both studies, different frying temperatures and time applied. 

However, the browning of market-purchased cereal-based products cannot be used as an indicator of the AA content probably because of the strong influence of raw material composition used and the manufacturing conditions on the AA content. This lack of correlation of AA with colour is not in agreement with the results reported by some authors for cookies, bread and crisp bread [29,30,31,32]. However, the research of the mentioned authors was carried out under controlled conditions and the same raw material was used. A clear correlation of acrylamide formation and browning colour was obtained for the asparagine-glucose model system [33]. On the other hand, the addition of asparagine increased the AA content but did not affect the colour [32,33]. In general, the relative correlation between browning and the acrylamide content occurs in model systems, and it is significant when ingredients and thermal-processing conditions are under control. The obtained results show that coping with the variability to find the appropriate kinetic models for the formation/degradation of acrylamide in commercially prepared food products is challenging. The food matrix is usually not homogeneous, which might hinder reactants ability to meet each other readily and it might also result in large variation in analytical measurements. Kinetic models established for food model systems cannot directly be applied in real foods because what takes place in the food itself, e.g., interaction among various compounds, changes in thermodynamic and the processing environment of the food, is normally neglected in the model systems. For this reason, information on the relationship between browning and acrylamide formation in food is often neglected and varies widely, in contrast to the results for model systems. Some researchers have reported that high-temperature, long-time treatment of foods is responsible for a great increase in AA content in foods, without causing significant changes in the colour or texture parameters [34,35]. On the other hand, in the case of some cereal-based products, it was stated that by producing lighter coloured and less baked products, the AA level could theoretically be reduced. In bread, the endpoint colour in most cases reflects the AA content. However, in some cases a darker colour may be associated with less AA, e.g., some breakfast cereals [36]. It is known that also moisture content, besides process temperature and the precursors in the raw material, is a key factor to limit AA formation.

In the present study, HMF content ranged widely from 17.1 mg/kg for French fries to 130 mg/kg for crisp bread (Table 1). It is known that thermal processing (roasting, toasting baking etc.), especially of carbohydrate-rich foodstuffs, has a huge impact on the HMF formation. The highest contents of HMF were reported for chicory, coffee, malt, dried fruits and caramel products with a range of 100–22,500 mg/kg product [14]. Cereal-based products such as breakfast cereals, bread, etc. due to their chemical composition, are also subjected to intensive HMF formation [37]. In the current study, the high contents of HMF were also determined in breakfast cereals, bread and biscuits (Table 1). The highest HMF content was found in crisp bread (Table 1), where the toasting temperature is generally more drastic than in traditional bread production and other cereal products such as biscuits. The low HMF contents in confectionery products (doughnuts, croissant and biscuits) in comparison to crisp bread was also reported by other authors [16,38,39]. Since potato products are not a significant source of simple sugars—important for the formation of HMF in caramelization and in Maillard reactions—the HMF content in these products was the lowest (Table 1). According to Zhang et al. [40], the HMF contents in potato products are very low (n.d. −9.5 mg/kg). 

The relationship between HMF levels and the colour are presented in Table 2. The correlation coefficients for HMF with “L*”, “a*” and “b*” values were significant (*p* < 0.05) for all market-purchased potato and cereal-based products. For cereal-based products, colour parameters were particularly highly correlated with the HMF content. The decrease in b* (blue-yellow) means that the yellow colour of products disappeared during the applied treatment while the HMF content increased. The L* parameter also showed a significant (negative) correlation with HMF content. During industrial production, cereal-based products darken as a result of carbohydrate transformation, including caramelisation. Based on the relationship between L* and the HMF content, the changes in the product colour can be linked with the thermal degradation and dehydration of sugars. In the tested cereal-based products, a significant (positive) correlation between a*parameters (red-green) and HMF formation was found. The high redness (a*) values may indicate an excessive caramelization of sugars and non-enzymatic browning reactions. The study showed that in terms of desired colour properties (lower content of HMF), higher L* and lower a* should be preferred. The results clearly indicate that colour information of bread, biscuits and breakfast cereals might be used to predict the HMF content in these groups of market-purchased products. Similar observations were made by Rufián-Henares et al. [37].

Both acrylamide and HMF can be formed in substantial concentrations in common heat-treated foods such as French fries, biscuits, bread, crisps and coffee (74–628 μg acrylamide/kg product and 0.5–22,500 mg HMF/kg product) [5]. The current study showed that the acrylamide content in potato products was correlated with the HMF content but there was no statistically significant correlation in commercial cereal-based products (Table 2). Rufián-Henares et al. [41] also showed no significant correlation between the contents of 5-hydroxymethylfurfural and browning with acrylamide levels in commercial breakfast cereals. The information on acrylamide and HMF formation mechanisms in real food products, i.e., food matrices more complex than model systems, is still limited. The specific amino acid route (especially asparagine) for the acrylamide formation and the caramelisation driven by fructose with or without glucose for the HMF formation are different in the reaction pathways leading to the formation of the two food contaminants under study. Thus, it is important to emphasise that HMF is not only generated by Maillard reactions, but it is also generated during the degradation of hexose and caramelization, which does not require the presence of an amino group. This might explain why the correlation between acrylamide and 5-hydroxymethylfurfural formation in a wide variety of market-purchased foods, particularly in the products with a high sugar content such as cereal-based foods, was not found. It seems that these differences in the formation of AA and HMF from components present in some types of food have a significant impact on the lack or presence of a correlation between acrylamide and 5-hydroxymethylfurfural content in market-purchased food. The types of wheat flour (in terms of different concentrations of total reducing sugars and asparagine) and the level of sugar addition led to different contents of acrylamide and HMF. These results are in agreement with the conclusion of the “Acrylamide Toolbox 2013”, which states that asparagine concentration, not sugar concentration, is a key determinant of the acrylamide formation in cereal products. This means that the concentration of acrylamide in cereal products correlates with the concentration of asparagine in cereal grain/flour [42]. However, in the case of the HMF formation it is just sugar concentration, which is a key determinant of this compound’s formation in cereal products. The current results indicate that in the cereal-based products, the generation of HMF and acrylamide followed the caramelisation route and the specific amino acid route, respectively. For potato products, probably due to the greater uniformity of the raw material (in terms of concentrations of total reducing sugars and asparagine) and the lack of added sugars, HMF generation mainly followed the specific amino acid route (as in the case of AA). This difference may be the reason for the correlation between AA and HMF levels in potato products and the lack of correlation between both compounds in cereal-based products. 

## 4. Conclusions

The significance of the correlation between the HMF level, colour parameters and the AA amount depended on the type of food. The current study showed a significant correlation between acrylamide formation and colour parameters as well as the HMF formation in market-purchased potato products. Thus, the HMF level and colour information such as the degree of surface browning can be considered indicators of the acrylamide content in market-purchased potato products. Therefore, a colour analysis system adapted to processing lines may be used for online monitoring of quality changes in these products. No significant relationships between acrylamide contents and HMF or between acrylamide level and colour parameters were found in market-purchased cereal-based products. Therefore, these classical thermal-processing indicators will not be useful for online monitoring of acrylamide since they are not directly related to acrylamide content in commercial cereal-based products. 

## Figures and Tables

**Table 1:** Colour parameter values, acrylamide (AA) and 5-hydroxymethylfurfural (HMF) content in commercial products.

Products	AA (µg/kg)	AA Benchmark Levels ^1^ (μg/kg)	HMF (mg/kg)	L*	a*	b*
French fries sold as ready to eat	377 ± 86 ^bc^	500	17.1 ± 4.0 ^d^	66.1 ± 2.4 ^a^	3.7 ± 1.3 ^c^	18.0 ± 0.3 ^a^
Potato crisps	546 ± 68 ^a^	750	46.3 ± 6.0 ^c^	60.8 ± 2.1 ^b^	5.8 ± 1.7 ^c^	16.2 ± 2.5 ^ab^
Soft wheat bread	55.0 ± 24 ^d^	50	82.2 ± 19 ^b^	52.2 ± 6.1 ^cd^	14.7 ± 4.9 ^b^	18.5 ± 3.3 ^ab^
Crisp wheat bread	312 ± 56 ^c^	350	130 ± 10 ^a^	48.9 ± 3.8 ^d^	27.1 ± 2.2 ^a^	15.1 ± 1.7 ^b^
Breakfast cereals	403 ± 110 ^abc^	300	69.0 ± 15 ^b^	59.2 ± 3.9 ^bc^	10.5 ± 2.4 ^b^	20.6 ± 2.9 ^a^
Biscuits	523 ± 95 ^ab^	350	92.1 ± 25 ^b^	50.7 ± 4.8 ^cd^	17.3 ± 5.9 ^b^	19.4 ± 2.1 ^a^

Data are expressed as mean values ± standard deviations (SDs) of ten independent samples (*n* = 10). ^1^ Acrylamide benchmark levels according to Commission Recommendation 2017/2158/EU [21]. Mean values in columns with the same letter are not significantly different (*p* < 0.05). L*, a* and b*: components of surface colour (L* represents lightness, positive a* = red, negative a* = green, positive b* = yellow and negative b* = blue).

**Table 2:** Correlations between acrylamide (AA), 5-hydroxymethylfurfural (HMF) and colour parameters in commercial products.

Products	Parameters	AA	HMF
French fries sold as ready to eat	HMF	0.478 *	1.000
	“L*”	−0.835 *	−0.189 *
	“a*”	0.825 *	0.322 *
	“b*”	0.386 *	−0.445 *
Potato chips	HMF	0.322 *	1.000
	“L*”	−0.384 *	−0.559 *
	“a*”	0.225 *	0.245 *
	“b*”	0.786 *	−0.578 *
Soft wheat bread	HMF	0.178	1.000
	“L*”	−0.185	−0.741 *
	“a*”	0.159	0.422 *
	“b*”	0.123	0.879 *
Crisp wheat bread	HMF	0.151	1.000
	“L*”	−0.086	−0.779 *
	“a*”	0.069	0.347 *
	“b*”	0.048	−0.785 *
Breakfast cereals	HMF	0.107	1.000
	“L*”	−0.135	−0.899 *
	“a*”	0.125	0.647 *
	“b*”	0.066	−0.988 *
Biscuits	HMF	0.115	1.000
	“L*”	−0.148	−0.855 *
	“a*”	0.075	0.401 *
	“b*”	0.106	−0.940 *

* Correlation coefficients statistically significant at *p* < 0.05. L*, a* and b*: components of surface colour (L* represents lightness, positive a* = red, negative a* = green, positive b* = yellow and negative b* = blue).
